# The Associations of Parents’ Psychological Well-Being and Resilience with Early Childhood Development

**DOI:** 10.3390/children13060735

**Published:** 2026-05-25

**Authors:** Şeyma Şimşirgil Kara, Kübra Gümüş, Çetin Çoban, Huriye Demet Cabar

**Affiliations:** 1 Department of Child Development, Faculty of Health Sciences, Sinop University, 57000 Sinop, Turkey; ccoban@sinop.edu.tr; 2 Department of Nursing, Faculty of Health Sciences, Sinop University, 57000 Sinop, Turkey; kgumustekin@sinop.edu.tr

**Keywords:** early childhood development, psychological well-being, psychological resilience, social-emotional development

## Abstract

Objective: This study examined the relationships between the developmental stages of children aged 6–72 months and their parents’ psychological resilience and psychological well-being. Method: The study was designed as a descriptive and correlational survey. The sample included a total of 184 children aged 6–72 months, as well as their parents, who visited Sinop Atatürk State Hospital. Data were collected face-to-face using the Personal Information Form, the Early Developmental Stages Inventory (EGE), the Psychological Well-being Scale (PWBS), and the Psychological Resilience Scale for Adults (PRS). The data were analyzed using the independent samples *t*-test, Pearson correlation coefficient, and simple and multiple linear regression analyses. Results: According to the study’s results, most children (92.4%) were found to be at a normal level in terms of overall development; importantly, personal-social skills had the highest rate of developmental delay (19.0%). Comparative analyses showed that the psychological resilience and psychological well-being scores of parents of children with typical development were higher than those of parents of children with developmental delays. Correlation analysis revealed a moderate positive relationship (r = 0.330, *p* < 0.001) between the Early Developmental Stages Inventory score and the Psychological Well-being Scale, and a weak positive relationship (r = 0.154, *p* < 0.05) between the Psychological Resilience Scale and the Early Developmental Stages Inventory score. Regression analyses suggest that psychological well-being and resilience are associated with child development outcomes. Discussion and Conclusions: The findings of this study indicate that there are meaningful associations between parents’ psychological well-being and resilience levels and children’s early developmental outcomes. Children whose parents reported higher psychological well-being and resilience tended to demonstrate more favorable developmental profiles in several developmental domains. These results suggest that parental psychological characteristics may be relevant factors associated with early childhood developmental outcomes and should be interpreted within broader ecological and contextual frameworks.

## 1. Introduction

Early childhood is a critical developmental period in which biological, environmental, and psychosocial factors interact to shape long-term cognitive, emotional, and social outcomes. Among these factors, the family environment, particularly parental characteristics, plays a supportive role in determining developmental trajectories [1]. Parents create the primary environment in which young children experience emotional regulation, social interaction, and early learning processes.

Parents’ psychological well-being is identified as a factor closely linked to the quality of care. Higher levels of well-being are associated with more responsive, sensitive, and supportive parenting behaviors, which in turn support positive developmental outcomes in children [2,3]. Conversely, parents’ psychological issues—including stress, anxiety, and depressive symptoms—have been linked to impairments in children’s cognitive, social-emotional, and behavioral development [4]. These effects are particularly pronounced during early childhood, which is a period when neuroplasticity is at its peak and children are highly sensitive to environmental influences [1].

In addition to psychological well-being, parental resilience—defined as the capacity to adapt positively to stress and challenges—has emerged as a key protective factor within the family system [5]. Resilient parents are more likely to maintain emotional stability, employ effective coping strategies, and provide consistent care despite challenging life circumstances, thereby creating a safer and more predictable environment for their children [5,6].

Despite the growing literature on the effects of parental mental health and child development, existing studies have generally examined psychological well-being and resilience separately or focused on limited developmental outcomes. Comprehensive research investigating the combined and comparative contributions of these parental factors to various domains of early childhood development, particularly in children aged 6–72 months, remains lacking.

Furthermore, relatively few studies have evaluated these relationships within a multivariable framework that simultaneously considers psychological, developmental, and educational factors together.

Therefore, the aim of this study is to examine the relationship between parents’ psychological well-being, resilience, and early childhood development outcomes and assess the predictive roles of these variables across different developmental domains.

Research Question:

How are parents’ psychological well-being and resilience related to the early childhood development of children aged 6–72 months?

## 2. Literature Review

### 2.1. Parents’ Psychological Well-Being and Child Development

Parents’ psychological well-being is a multidimensional concept encompassing emotional balance, life satisfaction, and positive psychological functioning [2]. Numerous studies indicate that parents with higher levels of psychological well-being are more likely to exhibit sensitive parenting behaviors that support children’s emotional regulation and social competence [3].

Conversely, impaired psychological well-being in parents has been associated with reduced parental sensitivity and less effective parent–child interactions. Empirical studies have shown that parental depression, anxiety, and chronic stress are linked to delays in language development, difficulties in behavioral regulation, and poorer social-emotional outcomes during early childhood [4,7], highlighting the critical role of parental mental health as a determinant of child development outcomes.

### 2.2. Parental Resilience as a Protective Factor

Resilience refers to an individual’s ability to successfully adapt to challenges and maintain functioning despite stress [5]. In the context of parenting, resilience influences how individuals cope with caregiving challenges and environmental stressors.

Compared to the well-established literature on psychological well-being, the evidence on parental resilience is less developed. Parental resilience is generally associated with a lower risk of child psychopathology and higher prosocial development, particularly when parents are able to maintain the quality of care despite stress [8,9,10].

Resilient parents tend to exhibit adaptive coping strategies, emotional regulation, and consistent caregiving behaviors, which contribute to a supportive family environment [6]. These factors have been linked to improved developmental outcomes in children, particularly in the social-emotional and behavioral domains.

At the same time, compared to psychological well-being, empirical evidence regarding parental resilience and its direct effects on many areas of early childhood development remains limited; this highlights the need for further research.

### 2.3. Early Childhood Development and Multidimensional Assessment

Early childhood development encompasses many interrelated areas, such as communication, motor skills, problem-solving abilities, and social-emotional functioning [11]. These areas develop in parallel and are influenced by both biological maturation and environmental factors.

Developmental screening tools, such as the Ages and Stages Questionnaire (ASQ), provide structured methods for assessing developmental progress and identifying potential delays [8]. Research indicates that social-emotional development is particularly sensitive to environmental influences, including parents’ mental health and the quality of care [12].

### 2.4. Research Gaps and the Study’s Contribution

Although previous studies have identified relationships between parents’ mental health and child development, certain limitations remain. First, psychological well-being and resilience are often examined independently without considering their combined effects. Second, many studies focus on specific areas of development rather than adopting a multidimensional perspective. Third, evidence from various clinical populations remains limited.

Furthermore, a substantial body of the literature relies on cross-sectional designs, which limits the ability to draw conclusions regarding directionality or causal relationships [1]. In addition, previous studies have varied considerably in terms of methodological approaches, developmental domains examined, and population characteristics. While some studies have focused primarily on socio-emotional outcomes, others have examined cognitive or behavioral domains separately, making direct comparisons difficult. Moreover, parental psychological well-being and resilience have generally been evaluated independently rather than within the same analytical framework. These methodological differences may partially explain inconsistencies in the literature and limit the development of a more integrative understanding of early childhood development [11,12].

Unlike many previous studies, the present study simultaneously examines psychological well-being, resilience, parental education, and multiple developmental domains within a single multivariable framework. By integrating psychological and structural variables together, this study aims to provide a broader ecological perspective on factors associated with early childhood development.

This study addresses these gaps by simultaneously examining parents’ psychological well-being and resilience in relation to multiple developmental domains in children aged 6–72 months. By presenting an integrative analysis, it contributes to a more comprehensive understanding of the role of parental psychological factors in early childhood development.

Unlike many previous studies that examined parental psychological well-being or resilience separately and focused on isolated developmental outcomes, the present study evaluates these variables simultaneously across multiple developmental domains within a single multivariable framework. In addition, by incorporating both psychological and educational variables into the same analytical model, the study adopts a broader ecological perspective on factors associated with early childhood development. This integrative approach may contribute to a more comprehensive understanding of how parental characteristics interact with developmental processes during early childhood.

Recent reviews have highlighted conceptual gaps in child well-being research, particularly the lack of integrative models that account for developmental, contextual, and family-level factors simultaneously [13]. Similarly, differences in measurement tools and cultural contexts have limited the generalizability of findings across populations.

When these limitations are considered together, it becomes evident that there is a need for studies that adopt a more comprehensive and integrative approach. This study addresses this gap by examining parents’ psychological well-being and resilience in relation to multiple developmental domains simultaneously within the same analytical framework, thereby offering a more holistic understanding of early childhood development.

## 3. Materials and Methods

### 3.1. Study Design and Setting

This study was designed as a cross-sectional, descriptive, and correlational study. Given the cross-sectional design, all findings are interpreted as associations, not causal relationships. Data were collected from parents of children aged 6–72 months who visited the pediatrics outpatient clinic at Sinop Atatürk State Hospital between January 2025 and June 2025.

### 3.2. Ethical Considerations

Ethical approval was obtained from the Sinop University Human Research Ethics Committee (Approval No.: 2024/268, Date: 27 September 2024). The research was conducted in accordance with the provisions of the Declaration of Helsinki.

### 3.3. Participants and Sampling Procedure

The target population consisted of approximately 1200 children who visited the pediatric outpatient clinic during the study period. The sample size was calculated using G*Power software(version 3.1.9.7) with a 95% confidence level and a 5% margin of error, and the minimum required sample size was determined to be 184 participants [14].

A convenience sampling method was used, and eligible participants were approached consecutively during routine clinical visits. Of the 210 parents invited to participate, 184 agreed and completed the study, resulting in a response rate of 87.5%.

### 3.4. Inclusion and Exclusion Criteria

Inclusion criteria:

- Parents of children aged 6–72 months;
- Visited the pediatric outpatient clinic during the study period;
- Ability to understand and respond to the questionnaires;
- Informed consent obtained.

Exclusion criteria:

- Children with severe neurological disorders or diagnosed developmental disabilities;
- Parents with known severe psychiatric disorders that could affect the reliability of responses;
- Incomplete or missing survey data.

### 3.5. Data Collection and Assessment Procedures

Data were collected through face-to-face interviews conducted in a private examination room in a clinical setting. Each assessment lasted approximately 15–20 min.

Parents completed the following measures:

- Structured sociodemographic questionnaire;
- Psychological Well-Being Scale (PWBS) [15];
- Psychological Resilience Scale (PRS) [16].

Children were assessed using the Early Developmental Stages Inventory (EDSI), which covers the areas of child development, communication, motor skills, problem solving, and social-emotional development [17].

All assessments were conducted by trained researchers to ensure consistency and minimize measurement bias.

### 3.6. Statistical Analysis and Rationale

Statistical analyses were performed using SPSS version 30.0. Descriptive statistics were presented as mean ± standard deviation for continuous variables and frequency (%) for categorical variables.

The normality of data distribution was assessed using skewness and kurtosis values. Since all variables showed an approximately normal distribution, parametric tests were applied.

Independent samples *t*-tests were used to compare group differences. Pearson correlation analysis was performed to examine the relationships between variables.

Simple and multiple linear regression analyses were conducted to assess the predictive effects of parents’ psychological well-being and resilience on early childhood development. These methods were selected in accordance with the study’s objectives, which aimed to evaluate both the correlational relationships and predictive relationships among continuous variables. Child age and parental education were included in the multivariable model because these variables are consistently identified in the literature as important factors associated with early childhood development and were available within the study dataset.

A *p*-value of <0.05 was considered statistically significant.

## 4. Results

Of the 184 children participating in the study, 52.2% were girls and 47.8% were male. Most respondents were mothers (88.0%), while 12.0% were fathers. Regarding parents’ educational levels, 65.8% were university graduates, 24.5% were high school graduates, 7.6% were middle school graduates, and 2.2% were elementary school graduates. During the form-filling process, 94.0% of parents did not receive assistance, while only 6.0% did. The mean age of the children was 38.61 months (SD = 19.65), ranging from 10 to 69 months. These findings indicate that the sample primarily consists of highly educated mothers and represents an age group that includes the early childhood period. The demographic characteristics of the participants and children are presented in Table 1.

The distribution of developmental levels according to the subscales of the Early Developmental Stages Inventory is presented in Table 2.

Most children demonstrated age-appropriate development across all domains. Notably, developmental delays were most frequently observed in the personal-social domain (19.0%), followed by communication, gross motor, problem-solving, and fine motor domains.

Descriptive statistics and normality values for the Psychological Resilience and Psychological Well-Being Scales are presented in Table 3.

The Psychological Resilience Scale demonstrated high internal consistency (Cronbach’s α = 0.841), whereas the Psychological Well-Being Scale showed acceptable reliability (Cronbach’s α = 0.766). Skewness and kurtosis values for both scales were within acceptable ranges, supporting the assumption of normal distribution [18,19]. These findings indicate that both instruments demonstrated adequate psychometric reliability for the present study.

Table 4 presents the comparison of parental psychological resilience scores according to children’s developmental status. Parents of children with typical development had significantly higher psychological resilience scores in communication, gross motor, problem-solving, and personal-social domains compared to parents of children with developmental delays. Effect sizes ranged from moderate to large, with the strongest differences observed in gross motor and personal-social development. No between-group difference was observed in fine motor development.

Table 5 presents the comparison of parental psychological well-being scores according to developmental domains. Parents of typically developing children demonstrated significantly higher psychological well-being scores in overall development, fine motor development, and personal-social development. Despite this, no notable differences were observed in communication, gross motor, or problem-solving domains.

The correlations between early developmental stages, parental psychological resilience, and parental psychological well-being are presented in Table 6.

Correlation analyses demonstrated positive associations between parents’ psychological characteristics and children’s developmental status. A weak positive correlation was observed between psychological resilience and developmental scores, whereas psychological well-being showed a moderate positive correlation with developmental outcomes.

The simple linear regression analysis of parental psychological resilience predicting early de-velopmental level is presented in Table 7.

Psychological resilience explained only 2.4% of the variance in child development scores (R^2^ = 0.024). This modest effect size warrants cautious interpretation.

The simple linear regression analysis of parental psychological well-being predicting early developmental stage is presented in Table 8.

Psychological well-being explained 10.9% of the variance in child development scores (R^2^ = 0.109), suggesting a stronger association than resilience.

Table 9 presents the results of the multiple linear regression analysis examining factors associated with early developmental scores. The overall model demonstrated moderate explanatory power (Adj. R^2^ = 0.491), indicating that the included variables collectively explained approximately half of the variance in developmental scores. Among the variables included in the model, parental education emerged as the strongest predictor of developmental outcomes (β = 0.629; *p* < 0.001), suggesting that structural and educational resources may play a substantial role in early childhood development. Psychological well-being also remained positively associated with developmental scores, whereas psychological resilience and child age did not demonstrate independent associations in the multivariable model.

## 5. Discussion

This study examined the relationships between parents’ psychological well-being, resilience, and developmental outcomes in children aged 6–72 months. Overall, children with more favorable developmental profiles tended to have parents with higher levels of psychological well-being and resilience, highlighting the potential relevance of parental psychological characteristics within early developmental processes. These relationships should be interpreted within the dynamic and multidimensional nature of early childhood development, which is influenced by interacting psychological, social, and environmental factors.

In this study, although the majority of children (92.4%) generally demonstrated age-appropriate development, there were differences across developmental domains. Developmental delays were most frequently observed in the personal-social domain (19.0%), followed by communication, gross motor, problem-solving, and fine motor domains. This pattern is consistent with previous research indicating that social-emotional development is particularly sensitive to environmental influences and the caregiving context [12]. Social-emotional competencies, such as emotional regulation, empathy, and interpersonal skills, are shaped through early interactions, and children may be more sensitive to changes in the family environment [20].

The finding that the rates of developmental delay in communication, gross motor, fine motor, and problem-solving skills are similar (ranging from 12% to 15%) indicates that these developmental domains are closely interrelated and that a delay in one area may affect the others. It is known that developmental domains progress in parallel and interact dynamically, particularly during the first years of life [13]. From this perspective, comprehensive developmental screening approaches are essential for the early identification of potential risks and the simultaneous consideration of multiple domains. Furthermore, theoretical frameworks such as social-emotional learning emphasize the importance of integrated developmental processes, despite the limitations of measurement approaches [21].

The findings of this study also indicate that parental resilience is positively associated with children’s developmental status. Higher resilience scores were observed among parents of typically developing children, particularly in the areas of communication, motor skills, problem-solving, and personal-social skills, which is consistent with the previous literature indicating that resilient parents tend to exhibit more adaptive coping strategies, emotional regulation, and consistent caregiving behaviors [5,22]. Such characteristics may contribute to a more supportive and predictable family environment, which is associated with positive developmental outcomes in children. Despite this, the absence of a notable difference in fine motor skills suggests that this area may be influenced more by the child’s biological maturation processes than by environmental or parental psychological factors.

Similarly, the analyses demonstrated that parents’ psychological well-being is associated with children’s developmental outcomes. Parents of typically developing children, particularly in areas such as fine motor skills and personal-social development, reported higher psychological well-being scores. Previous studies have shown that higher levels of parental well-being are associated with more responsive and supportive parenting practices that may facilitate children’s cognitive and socio-emotional development [3,23]. Additionally, parents’ emotional stability and life satisfaction have been linked to children’s sense of security and social participation [24]. It has been reported that parents with high psychological resilience respond more flexibly to negative events in their lives and, as a result, provide their children with a safer, more predictable and supportive environment [5].

Correlation analyses further support these findings, demonstrating positive relationships between parents’ psychological characteristics and children’s early developmental status. The observed relationships suggest that both parental resilience and psychological well-being may function as supportive factors within the parent–child system. Nevertheless, the relatively modest effect sizes observed for psychological variables suggest that these factors likely represent only one component of a broader developmental system involving multiple interacting biological, familial, socioeconomic, and environmental determinants.

Regression analyses demonstrated that parents’ psychological well-being and psychological resilience are positively associated with child development. However, the explanatory power of these models is limited, suggesting that additional variables such as socioeconomic status, parental education level, and environmental conditions may also play important roles. This finding is consistent with the existing literature, which emphasizes the multifactorial nature of early childhood development [4,25]. Notably, parental education emerged as the strongest variable in the multivariable model, suggesting that structural and educational resources may exert broader and more stable influences on early childhood development than individual psychological characteristics alone.

Importantly, the findings of this study should be interpreted within the limitations of its cross-sectional design. The observed associations do not allow for conclusions regarding causality or directionality. Rather than parental characteristics directly influencing child outcomes, it is possible that more positive developmental profiles in children are associated with higher parental well-being. Indeed, previous studies have demonstrated that there are reciprocal and dynamic interactions between parenting behaviors and child development [23,26]. Therefore, longitudinal and multivariable research designs are needed to clarify the directionality of these relationships and the underlying mechanisms. Additionally, unmeasured contextual factors such as family socioeconomic conditions, parental employment, access to educational resources, and broader caregiving environments may also have contributed to the observed developmental differences. Therefore, the associations identified in this study should be interpreted within a broader ecological and contextual framework rather than as isolated psychological effects.

In this study, the fact that the data were obtained primarily through self-report measures and largely from mothers should be considered a limitation, as self-report data could be subject to social desirability and subjective evaluation bias. Additionally, the fact that the sample consists of a highly educated and relatively homogeneous group recruited from a single center may limit the generalizability of the findings to different socioeconomic, cultural, and environmental contexts. Therefore, the potential effects of these contextual variables should be taken into account when interpreting the results.

In particular, the emergence of parental education as the strongest variable supports the notion that early childhood development is closely linked not only to psychological factors but also to socioeconomic and cognitive resources. This finding is consistent with studies in the literature that emphasize the important role of parental education level in children’s cognitive and socio-emotional development [4].

The persistence of psychological well-being as an independent predictor suggests that parents’ emotional states may be related to child development. However, it is clear that this relationship has a relatively limited effect size and must be evaluated in conjunction with other variables in the model. Conversely, the loss of significance for psychological resilience in the multiple regression model suggests that the relationships observed for this variable may not be independent of other factors. This indicates that the relationships observed in previous analyses may have been influenced by potential confounding variables.

The present study contributes to the literature by evaluating parental psychological well-being and resilience together across multiple developmental domains rather than focusing on isolated developmental outcomes alone.

Taken together, these findings suggest that parents’ psychological well-being and resilience may be associated with early childhood developmental outcomes within a broader ecological framework. Rather than functioning as direct determinants, these variables appear to interact with multiple structural, socioeconomic, and environmental factors. Future longitudinal and multivariable studies are needed to clarify these complex relationships and better identify the mechanisms underlying child development.

## 6. Conclusions

This study demonstrates that there are positive and statistically significant relationships between parents’ psychological well-being and resilience and early childhood development. However, the results of the multiple regression analysis reveal that these relationships may vary when all variables are considered together. This study reinforces the multifactorial nature of early childhood development and highlights the importance of considering psychological, structural, and contextual factors together.

### 6.1. Recommendations

Given the cross-sectional design of this study, the following recommendations are tentative and hypothesis-generating rather than evidence-based conclusions.

If future longitudinal or experimental research confirms the observed associations, parent-focused interventions within early childhood support systems might be considered. Psycho-educational interventions aimed at improving parents’ emotional states, stress management skills, and resilience levels could be explored. Training programs for healthcare professionals, early childhood educators, and community programs may help them recognize parental stress and provide timely support or referrals. Clinical and community settings might benefit from adopting a holistic, family-centered approach that prioritizes the well-being of both parents and children. Additionally, emotional coping skills and parenting capacity could be developed through workshops, counseling, or digital formats, particularly in families exposed to environmental or social stressors. However, these suggestions require testing in randomized controlled trials or intervention studies before implementation.

### 6.2. Limitations

While this study presents valuable findings, certain limitations should be taken into account. First, the fact that the data were obtained from a single state hospital located in Sinop Province (Sinop Atatürk State Hospital) means that the sample reflects a specific socioeconomic and cultural context, thereby limiting the generalizability of the findings. Additionally, potentially important socioeconomic variables such as household income, employment status, and broader socioeconomic indicators were not available in the dataset and therefore could not be included in the regression models. The absence of these variables may have limited the explanatory capacity of the multivariable analyses. The data were collected using self-report measures based on parents’ own statements, which may have led to social desirability and subjective assessment bias. Furthermore, due to the study’s cross-sectional design, it is not possible to draw definitive conclusions regarding the causal nature and direction of the observed relationships. In this context, it should not be overlooked that while parental characteristics influence child development, children’s developmental characteristics may also influence parents’ psychological well-being. Therefore, the results should be interpreted not as reflecting a causal relationship but as indicating potential associations between the variables.

### 6.3. Future Directions

Future studies may benefit from using longitudinal or intervention-based research designs to better clarify potential causal relationships and explore how parental resilience may be associated with developmental outcomes in children. Further research may also examine which specific resilience-related factors, such as problem-solving abilities, emotional regulation, or social support, are more closely linked to different developmental domains. In addition, family-centered programs tailored to socioeconomic background and contextual risk factors may be explored and evaluated in future studies. Larger-scale and longitudinal investigations may further contribute to understanding the most effective approaches for supporting parental capacity and child development. A better understanding of the mechanisms linking parental psychological well-being and child development may also contribute to informing future early intervention strategies and family support policies.

## Figures and Tables

**Table 1:** Demographic characteristics of participants.

		*n*	%
Child’s Gender	Female	96	52.2
	Male	88	47.8
Relationship to Child	Mother	162	88.0
	Father	22	12.0
Educational Status	Elementary school	4	2.2
	Middle School	14	7.6
	High School	45	24.5
	University degree or higher	121	65.8
Assistance in Completing Questionnaires	Yes	11	6.0
	No	173	94.0
		Mean ± SD	Lower–Upper Value
Child Age (months)		38.61 ± 19.65	10–69

**Table 2:** Distribution of developmental levels according to the subscales of the Early Developmental Stages Inventory.

		n	%
Early Developmental Stages Inventory	Delayed	14	7.6
	Normal	170	92.4
Communication	Delayed	27	14.7
	Normal	157	85.3
Gross Motor	Delayed	26	14.1
	Normal	158	85.9
Fine Motor	Delayed	22	12.0
	Normal	162	88.0
Problem-Solving	Delayed	25	13.6
	Normal	159	86.4
Personal-Social	Delayed	35	19.0
	Normal	149	81.0

**Table 3:** Descriptive statistics and normality values for the Psychological Resilience and Psychological Well-Being Scales.

	$\bar{x}$ ± SD	Minimum–Maximum Values	CA (α)	Skewness	Kurtosis
Psychological Resilience Scale	141.03 ± 16.24	104–167	0.841	−0.514	−0.687
Psychological Well-Being Scale	185.74 ± 12.66	154–209	0.766	−0.209	−0.380

$\bar{x}$ ± SD = mean ± standard deviation, CA (α) = Cronbach’s alpha.

**Table 4:** Comparison of Psychological Resilience Scale scores according to the subdimensions of the Early Developmental Stages Inventory.

		Psychological Resilience Scale					
		n	Mean	Standard Deviation	*t*	*p*	d
Early Developmental Stages Inventory	Delayed	14	132.36	5.99	2.099	0.037	0.584
	Normal	170	141.75	16.62			
Communication	Delayed	27	131.93	16.88	3.235	<0.001	0.674
	Normal	157	142.60	15.66			
Gross Motor	Delayed	26	127.50	14.45	4.860	<0.001	1.029
	Normal	158	143.26	15.46			
Fine Motor	Delayed	22	139.18	11.59	0.569	0.570	
	Normal	162	141.28	16.79			
Problem-Solving	Delayed	25	130.44	15.98	3.622	<0.001	0.779
	Normal	159	142.70	15.69			
Personal-Social	Delayed	35	130.69	13.71	4.393	<0.001	0.825
	Normal	149	143.46	15.87			

Independent samples *t*-test, *p* < 0.05.

**Table 5:** Comparison of Psychological Well-Being Scale scores according to the subdimensions of the Early Developmental Stages Inventory.

		Psychological Well-Being Scale					
		n	Mean	Standard Deviation	*t*	*p*	d
Early Developmental Stages Inventory	Delayed	14	171.21	0.97	4.719	<0.001	1.312
	Normal	170	186.94	12.43			
Communication	Delayed	27	182.67	15.88	1.371	0.172	
	Normal	157	186.27	12.00			
Gross Motor	Delayed	26	184.92	15.38	0.356	0.722	
	Normal	158	185.88	12.21			
Fine Motor	Delayed	22	180.09	12.54	2.257	0.025	0.513
	Normal	162	186.51	12.52			
Problem-Solving	Delayed	25	185.72	16.31	0.010	0.992	
	Normal	159	185.75	12.05			
Personal-Social	Delayed	35	180.97	14.81	2.514	0.013	0.472
	Normal	149	186.87	11.88			

Independent samples *t*-test, *p* < 0.05.

**Table 6:** Correlations between early developmental stages, parental psychological resilience, and parental psychological well-being.

		Early Developmental Stages Inventory	Psychological Resilience Scale	Psychological Well-Being Scale
Early Developmental Stages Inventory	r	1		
	*p*			
Psychological Resilience Scale	r	0.154 *	1	
	*p*	0.037		
Psychological Well-Being Scale	r	0.330 **	−0.111	1
	*p*	<0.001	0.134	

Pearson correlation test; * *p* < 0.05, ** *p* < 0.001.

**Table 7:** Simple linear regression analysis of parental psychological resilience predicting early developmental level.

						95% Confidence Interval	
	$\hat{\beta }$	$\mathrm{SE}\;\hat{\beta }$	Beta	*t*	*p*-Value	Lower Bound	Upper Bound
(Fixed)	1.569	0.170		9.218	<0.001	1.233	1.905
Psychological Resilience Scale	0.003	0.001	0.154	2.099	0.037	0.000	0.005
	R	R^2^	F	*p*			
	0.154	0.024	4.404	0.037			

Dependent variable: Early Development Stages Inventory.

**Table 8:** Simple linear regression analysis of parental psychological well-being predicting early developmental stage.

						95% Confidence Interval	
	$\hat{\beta }$	$\mathrm{SE}\;\hat{\beta }$	Beta	*t*	*p*-Value	Lower Bound	Upper Bound
(Fixed)	0.636	0.273		2.326	0.021	0.096	1.176
Psychological Well-Being Scale	0.007	0.001	0.330	4.719	<0.001	0.004	0.010
	R	R^2^	F	*p*			
	0.330	0.109	22.273	<0.001			

Dependent variable: Early Development Stages Inventory.

**Table 9:** Multiple linear regression analysis examining the effects of psychological resilience, psychological well-being, child’s age, and parental education on early developmental stage.

						95% Confidence Interval	
	$\hat{\beta }$	$\mathrm{SE}\;\hat{\beta }$	Beta	*t*	*p*-Value	Lower Bound	Upper Bound
(Constant)	0.081	0.259		0.313	0.755	0.430	0.592
Psychological Resilience Scale	0.001	0.001	0.087	1.610	0.109	0.000	0.003
Psychological Well-Being Scale	0.003	0.001	0.154	2.700	0.008	0.001	0.006
Child’s Age (months)	0.000	0.001	0.011	0.178	0.859	0.001	0.002
Parent Education	0.229	0.022	0.629	10.201	<0.001	0.184	0.273
	R^2^	Adj. R^2^	F	*p*			
	0.502	0.491	45.060	<0.001			

## Data Availability

The data presented in this study are available on request from the corresponding author. The data are not publicly available due to privacy.
